# De novo* HNF4A*-associated atypical Fanconi renal tubulopathy syndrome

**DOI:** 10.1007/s40620-023-01666-0

**Published:** 2023-06-13

**Authors:** Rebecca Hudson, Natasha Abeysekera, Penny Wolski, Cas Simons, Leo Francis, Elizabeth Farnsworth, Bruce Bennetts, Chirag Patel, Siebe Spijker, Andrew Mallett

**Affiliations:** 1 Department of Renal Medicine, Royal Brisbane and Women’s Hospital, Herston, QLD Australia; 2 Department of General Surgery, Royal Brisbane and Women’s Hospital, Herston, QLD Australia; 3 Department of Diabetes and Endocrinology, Royal Brisbane and Women’s Hospital, Herston, QLD Australia; 4 Centre for Population Genomics, Garvan Institute of Medical Research, and University of New South Wales, Sydney, NSW Australia; 5 Centre for Population Genomics, Murdoch Children’s Research Institute, Melbourne, VIC Australia; 6 Anatomical Pathology, Pathology Queensland, Royal Brisbane and Women’s Hospital, Herston, QLD Australia; 7 Sydney Genome Diagnostics, Western Sydney Genetics Program, Children’s Hospital Westmead, Westmead, NSW Australia; 8 Specialty of Genomic Medicine, Faculty of Medicine and Health, University of Sydney, Sydney, NSW Australia; 9 Genetic Health Queensland, Royal Brisbane and Women’s Hospital, Herston, QLD Australia; 10 Department of Nephrology, Leiden University Medical Centre, Leiden, The Netherlands; 11 Department of Renal Medicine, Townsville Hospital and Health Service, Townsville University Hospital, 100 Angus Smith Drive, Douglas, QLD 4814 Australia; 12 Faculty of Medicine, James Cook University, Townsville, QLD Australia; 13 Faculty of Medicine, The University of Queensland, Herston, QLD Australia; 14 Institute for Molecular Bioscience, The University of Queensland, St Lucia, QLD Australia

**Keywords:** *HNF4A*, Atypical Fanconi Renal Tubulopathy Syndrome, Tubulopathy, De novo monogenic rare disease

## The Case

A 10-year-old male presented with hypophosphataemic rickets requiring subsequent multi-level orthopaedic procedures, Fanconi renotubulopathy (hypophosphataemia, phosphaturia, glycosuria and aminoaciduria in the absence of renal tubular acidosis and hypouricaemia) and progressive chronic kidney disease with bland urinary sediment and albuminuria (24 mg/mmol). He experienced hypocalcaemia in the absence of hypercalciuria, though his other serum and urine electrolytes were unremarkable (Stable 1). In addition to a past history of a surgically repaired ventricular septal defect (VSD) at 5 years, he suffered growth delay refractory to growth hormone therapy. This was complicated by hyperglycaemia at 15 years, which improved upon cessation of growth hormone therapy (normal fasting glucose 5.1 mmol/L and HbA1c 5.2%) but intermittent hyperglycaemia persisted. His development was otherwise normal. Notably, he was not macrosomic at birth nor suffered neonatal hyperinsulinism.

He has two siblings, a brother and sister; his sister has ureteric reflux receiving ureteroplasty at 6 years, and his parents have no history of kidney disease. His mother had no phenotypic nor genetic findings consistent with Dent disease.

There were no abnormalities on kidney imaging, including absence of nephrocalcinosis. Liver biochemistry and imaging were unremarkable. Kidney biopsy showed normal glomeruli and vessels with only minor tubular epithelial cell changes. Electron microscopy showed mild morphological changes in mitochondria of the tubular epithelial cells (SFigs. 1, 2).

*CLCN5*/*OCRL* genetic analysis was unremarkable, and the patient was diagnosed initially with Dent disease-like proximal renal tubulopathy. At 22 years, the patient and his parents provided informed consent and underwent subsequent trio whole exome sequencing which identified the de novo heterozygous *HNF4A* p.R85W mutation (NM_175914.4: c.[187C > T];[ =] p.[(Arg63Trp)];[ =]). This result was confirmed in a clinically accredited laboratory and returned to the patient and family with further genetic counselling. Definitive multilevel orthopaedic lower limb surgeries were undertaken at 23 years (SFig. 3). The patient subsequently re-developed diabetes mellitus responsive to small doses of sulphonylurea at 24 years which was identified due to regular genomically-informed investigations.

## Lessons for the clinical nephrologist

**Fig. 1:**
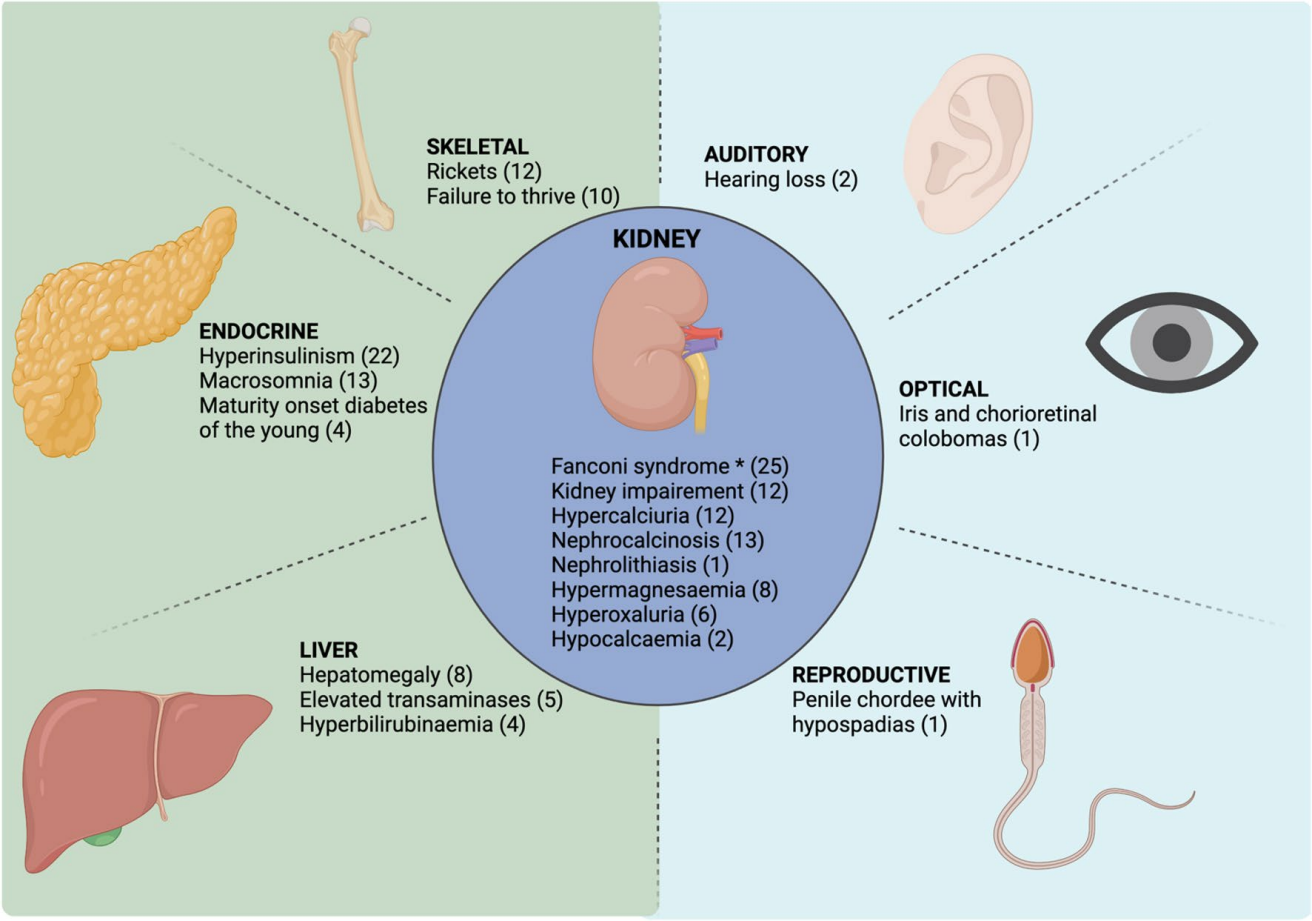
Reported renal and extra-renal phenotypes of the HNF4A p.R85W mutation. The major extra-renal phenotypes are located on the left highlighted in green, with the less common extra-renal phenotypes located on the right highlighted in blue. Phenotypic overlap within the clinical presentations of this HNF4A variant are discussed throughout this review. Created with BioRender.com. *Fanconi syndrome defined as hyperphosphaturia with hypophosphataemia, glycosuria with normal serum glucose, metabolic acidosis, low molecular weight proteinuria, aminoaciduria and hypouricaemia. The number of reported cases is located in brackets next to each phenotype

Heterozygous Hepatocyte Nuclear Factor 4A (*HNF4A)* mutations are known to cause hyperinsulinaemic hypoglycaemia and macrosomia in the neonatal period, in addition to the risk of MODY-1 [1–3]. Since 2012, the phenotypic spectrum associated with *HNF4A* mutations has been extended to include renal tubular dysfunction. Here we focus on the expanding kidney phenotype and complications of this heterozygous *HNF4A* p.R85W mutation (Fig. 1).

Fanconi renal tubulopathy syndrome (FRTS) is characterised by generalised proximal renal tubular dysfunction that results in the failure of glucose, low-molecular weight proteins, phosphate, bicarbonate and urate reabsorption [4, 5]. It is most commonly diagnosed during childhood, with common genetic causes being that of Dent disease (*CLCN5*) and Lowe syndrome (*OCRL*), however there has been an increase in the identification of novel genetic aetiologies [4]. Fanconi renal tubulopathy syndrome type 4 (Hepatocyte Nuclear Factor 4A) [FRTS4] expands the kidney phenotype to include hypercalciuria, relative hypocalcaemia, hypermagnesaemia, nephrocalcinosis and kidney impairment, and *HNF4A*-associated atypical FRTS (OMIM:FRTS4,#616,026). The *HNF4A* p.R85W mutation was first reported as a novel pathogenic variant in 2010 [2], presenting with hyperinsulinism that transitions to MODY-1 in the absence of kidney manifestations. The kidney phenotype associated with the *HNF4A* p.R85W mutation was first described in 2012 [6] in a case presentation of neonatal hyperinsulinism with features of Fanconi syndrome. This was further substantiated in a case series^7^ of six patients with features of Fanconi syndrome and nephrocalcinosis, later referred to as FRTS4. Since the aforementioned case series, there have been 17 cases in the literature reported with FRTS caused by this heterozygous *HNF4A* p.R85W mutation [5–9, S10−S17] (Table 1).

**Table 1:** Clinical phenotypes of patients with the *HNF4A* p.R85W mutation. Adapted from Liu et al. [5]

Patient No.	1	2	3	4	5	6	7	8
Family	I	II	II	II	III	IV	V	VI
HNF4A variant	p.R76W de novo	p.R76W *demonstrated inheritance*	p.R76W *demonstrated inheritance*	p.R76W *demonstrated inheritance*	p.R76W	p.R76W	p.R76W	p.R76W de novo
Age at presentation	NA	N	N	N	N	N	N	6 m
Sex at birth	F	F	F	M	F	F	F	F
Macrosomia	–	+	+	+	–	+	–	–
Hypoglycaemia	+	+	+	+	+	+	+	+
Diabetes (onset age)	–	–	–	–	+ 20y	+ 12y	–	NA
Electrolyte abnormality	Partial FS Hypophosphataemia, metabolic acidosis, glycosuria, amino aciduria	FS	FS	FS	FS	FS	FS	FS
Fanconi Syndrome (onset age)	+ 1y	+ 25y	+ 23y	+ N	+ 3y	+ 4y	+ 4y	+ 6 m
Liver Involvement (onset age)	+ 3 m	–	–	–	–	–	–	NA
Growth Delay	NA	+	+	+	+	+	+	NA
Rickets	+	+	NA	NA	NA	NA	NA	+
Nephrocalcinosis	NA	+	+	+	+	+	+	+
eGFR (ml/min)	NA	47	39	42	23	60	62	NA
References	Stanescu et al. [6]	Hamilton et al. [7]	Hamilton et al. [7]	Hamilton et al. [7]	Hamilton et al. [7]	Hamilton et al. [7]	Hamilton et al. [7]	Brichta [13]

Patient No.	1	2	3	4	5	6	7	8
Family	I	II	II	II	III	IV	V	VI
HNF4A variant	p.R76W de novo	p.R76W *demonstrated inheritance*	p.R76W *demonstrated inheritance*	p.R76W *demonstrated inheritance*	p.R76W	p.R76W	p.R76W	p.R76W de novo
Age at presentation	NA	N	N	N	N	N	N	6 m
Sex at birth	F	F	F	M	F	F	F	F
Macrosomia	–	+	+	+	–	+	–	–
Hypoglycaemia	+	+	+	+	+	+	+	+
Diabetes (onset age)	–	–	–	–	+ 20y	+ 12y	–	NA
Electrolyte abnormality	Partial FS Hypophosphataemia, metabolic acidosis, glycosuria, amino aciduria	FS	FS	FS	FS	FS	FS	FS
Fanconi Syndrome (onset age)	+ 1y	+ 25y	+ 23y	+ N	+ 3y	+ 4y	+ 4y	+ 6 m
Liver Involvement (onset age)	+ 3 m	–	–	–	–	–	–	NA
Growth Delay	NA	+	+	+	+	+	+	NA
Rickets	+	+	NA	NA	NA	NA	NA	+
Nephrocalcinosis	NA	+	+	+	+	+	+	+
eGFR (ml/min)	NA	47	39	42	23	60	62	NA
References	Stanescu et al. [6]	Hamilton et al. [7]	Hamilton et al. [7]	Hamilton et al. [7]	Hamilton et al. [7]	Hamilton et al. [7]	Hamilton et al. [7]	Brichta [13]

Patient No.	9	10	11	12	13	14	15	16
Family	VII	VIII	IX	X	XI	XII	XIII	XIII
HNF4A variant	p.R76W de novo	p.R76W de novo	p.R76W de novo	p.R63W de novo	p.R63W	p.R63W de novo	p.R63W de novo	p.R63W *demonstrated* *inheritance*
Age at presentation	6 m	N	N	N	N	N	N	N
Sex at birth	M	M	M	M	M	M	F	M
Macrosomia	+	+	+	+	–	+	NA	–
Hypoglycaemia	–	+	+	+	+	+	+	+
Diabetes (onset age)	NA	–	–	–	–	–	–	–
Electrolyte abnormality	FS	FS	FS	Partial FS Phosphaturia, aminoaciduria	Partial FS Hypophosphataemia, phosphaturia	Partial FS Hypophosphataemia, metabolic acidosis, glycosuria, phosphaturia, aminoaciduria	FS	FS
Fanconi Syndrome (onset age)	+ 6 m	+ 4 m	+ 8 m	+ N	+ N	+ 18 m	+ 3y	+ N
Liver Involvement (onset age)	NA	–	+ 7 m	+ N	–	+ 6 m	+ N	–
Growth Delay	+	NA	+	NA	NA	+	+	NA
Rickets	+	+	–	NA	NA	+	+	–
Nephrocalcinosis	–	–	–	–	–	NA	+	NA
eGFR (ml/min)	NA	NA	NA	NA	NA	NA	47	NA
References	Brichta [13]	Numakura [14]	Numakura [14]	Improda [15]	Improda [15]	Clemente et al. [7]	Walsh [16]	Walsh [16]

Patient No.	17	18	19	20	21	22	23	24	25
Family	XIV	XV	XV	XVI	XVI	XVII	XVIII	XIX	XX
HNF4A variant	p.R63W de novo	p.R63W	p.R63W *demonstrated inheritance*	p.R85W	p.R85W *demonstrated inheritance*	p.R85W de novo	p.R63W de novo	p.R63W	p.R85W de novo
Age at presentation	3 m	3y	N	NA	2 days	3y	N	N	10y
Sex at birth	F	F	NA	F	M	M	M	M	M
Macrosomia	NA	NA	NA	–	–	NA	–	–	–
Hypoglycaemia	+	+	+	+	+	NA	+	+	–
Diabetes (onset age)	–	+ gestational	NA	+ 13y	NA	–	–	–	Drug related 10-15yrs (Growth Hormone), re-emerged at 26yrs
Electrolyte abnormality	FS	FS	NA	Partial FS	Partial FS Hypophosphataemia, glycosuria, phosphaturia, proteinuria	Partial FS Glycosuria, aminoaciduria	FS Nil specifics	FS Nil specifics	Partial FS Hypophosphataemia, glycosuria, aminoaciduria
Fanconi Syndrome (onset age)	+ 5y	+ 3y	Confirmed Mutation	+	+ N	+ 3y	+	+	10y
Liver Involvement (onset age)	+ 3 m	–	NA	+	+ N	NA	+	–	–
Growth Delay	+	NA	NA	+	+	+	+	+	+
Rickets	+	+	NA	+	–	+	NA	NA	+
Nephrocalcinosis	+	+	NA	+	–	+	NA	NA	**-**
eGFR (ml/min)	56	21	NA	NA	NA	63	NA	NA	51
Reference	Liu et al. [5]	Anyiam et al. [8]	Anyiam et al. [8]	Sheppard [10]	Sheppard [10]	Duan [11]	McGlacken-Byrne [12]	McGlacken-Byrne [12]	*This case report*

*NA* not available, *N* Neonatal, *m* month, *F* female, *M* male, *y* year, + yes, – no, *eGFR* estimated glomerular filtration rate, *FS* Fanconi syndrome

Renal Fanconi syndrome as defined either by the journal, or electrolyte disturbance meeting criteria; hyperphosphaturia with hypophosphataemia, glycosuria with normal serum glucose, metabolic acidosis, low molecular weight proteinuria, aminoaciduria and hypouricaemia

Those without all features of FS or defined as FS are labelled partial FS with specific electrolyte disturbances listed

The pancreatic beta cell phenotype of *HNF4A* mutations is well documented [1–3], however, a kidney phenotype was not recognised until identification of a specific heterozygous missense mutation in the DNA-binding domain of *HNF4A* (p.R85W) [6, 7]. In humans and rodents, *HNF4A* mRNA undergoes extensive alternating splicing^S18^, which has resulted in some confusion in the literature with the same mutation being referred to as p.R63W [5, 9, S10, S12], p.R76W [6, 7, S11] or p.R85W [S19]. This is due to the mutation having different names depending on which spliced isoform is used as a reference [4], but all pertaining to the same genetic variant and clinical syndrome. The HNF4A spliced isoform that contains p.R85W is substantially expressed in the kidney tubules, as such, it has been proposed that p.R85W be the reference sequence for FRTS4 [4]. The *HNF4A* p.R85W mutation occurs within a DNA binding domain, mutations of which have been thought to involve modified DNA binding via altered transcriptional activity that is expressed in the liver, pancreas and kidney tubules [S20]. Further, this variant is reported to exhibit a dominant-negative effect as opposed to haploinsufficiency that has been observed with other *HNF4A* variants [S21]. FRTS only affects the proximal tubule, implying that the function of HNF4A is specific to the proximal tubule [S22]. Marable et al. [S23] hypothesised that the expression of HNF4A is critical in the maintenance of transporters within the kidney proximal tubules, as is supported by *HNF4A* knockout mouse models that demonstrate FRTS, kidney tubular dysgenesis and nephrocalcinosis [S23].

The clinical presentation and spectrum of FRTS4 caused by this heterozygous *HNF4A* pathogenic missense variant has been expanded [5–9], [S10−S12, S14−S17] following the initial publication in 2012 [6]. Neonatal patients can present with macrosomia and hyperinsulinism, and some develop diabetes in adolescence or early adulthood. Patients present with atypical FRTS with electrolyte disturbances, acidaemia, failure to thrive, hypophosphataemic rickets in childhood, osteomalacia in adulthood, hypercalciuria, nephrocalcinosis and kidney impairment. Other features include liver involvement with elevated transaminases, hepatomegaly, jaundice and liver cysts. Hearing loss has been reported in two cases, with one case reported to have multiple congenital anomalies such as penile chordee with hypospadias and iris and chorioretinal colobomas.

The phenotype of our patient harbouring the heterozygous *HNF4A* p.R85W mutation is generally similar to previously reported cases. Whilst initially experiencing hyperglycaemia related to growth hormone therapy which resolved on cessation of treatment at 15 years, sulphonylurea-sensitive diabetes consistent with MODY-1 re-emerged in early adulthood. His predominant features were those of atypical FRTS with electrolyte disturbances, hypophosphataemic rickets, low molecular weight proteinuria and progressive kidney impairment, though not hypercalciuria or nephrocalcinosis. Our patient also had a VSD repaired in early life; there has been one further case reported of a VSD in a patient affected by the *HNF4A* p.R85W mutation [S16]. Cardiac anomalies are common in the general population and so this may not be causal, but it has been reported that HNF4A is expressed somewhat in adult myocytes [S24]. Our case reconfirms the kidney phenotype of FRTS4 in addition to MODY-1 in young adulthood and to potential cardiac structural anomalies. It also highlights that de novo presentations should be considered in phenotypes usually associated with autosomal dominant inheritance but without apparent family history, and emphasizes the importance of a genetic diagnosis to ensure long-term surveillance and management of both kidney and extra-renal manifestations. Lastly, our case highlights how selective application of trio genomic sequencing can have diagnostic utility, especially after an initially negative singleton genomic test or gene panel in a clinical scenario of heightened suspicion of a monogenic diagnosis.

In summary, FRTS4 caused by the specific heterozygous *HNF4A* p.R85W mutation manifests as Fanconi syndrome with calcium and magnesium dysregulation, nephrocalcinosis. kidney impairment, and extra-renal phenotypes including rickets and MODY-1. FRTS4 is likely underdiagnosed though nephrologists play a significant role in the diagnosis and care of affected patients with complex kidney and extra-renal manifestations. This case highlights the importance of confirming a molecular diagnosis in affected patients, including those with a phenotype approximating Dent Disease without an identified causative genotype (Table 2).

**Table 2:** Teaching points

Atypical Fanconi Renal Tubulopathy Syndrome (FRTS4) can be due to the heterozygous *HNF4A* p. p.R85W mutation
FRST4 can have endocrine, skeletal, hepatic and other extra-renal phenotypes
FRST4 can phenocopy other forms of Fanconi renal tubulopathy syndrome such as Dent disease and Lowe syndrome
De novo presentations should be considered in phenotypes usually associated with autosomal dominant inheritance but without apparent family history

### Supplementary Information

Below is the link to the electronic supplementary material.

Supplementary file1 (DOCX 1156 KB)

## Declarations

### Human and animals rights

This study included human participants and has been performed in accordance with the ethical standards of the Declaration of Helsinki and its later amendments.

## References

[1] Kapoor RR, Locke J, Colclough K (2008). Persistent hyperinsulinemic hypoglycemia and maturity-onset diabetes of the young due to heterozygous HNF4A mutations. Diabetes.

[2] Flanagan SE, Kapoor RR, Mali G (2010). Diazoxide-responsive hyperinsulinemic hypoglycemia caused by HNF4A gene mutations. Eur J Endocrinol.

[3] Yamagata K, Furuta H, Oda N (1996). Mutations in the hepatocyte nuclear factor-4alpha gene in maturity-onset diabetes of the young (MODY1). Nature.

[4] Lemaire M (2021). Novel Fanconi renotubular syndromes provide insights in proximal tubule pathophysiology. Am J Physiol Renal Physiol.

[5] Liu J, Shen Q, Li G (2018). HNF4A-related Fanconi syndrome in a Chinese patient: a case report and review of the literature. J Med Case Rep.

[6] Stanescu DE, Hughes N, Kaplan B (2012). Novel presentations of congenital hyperinsulinism due to mutations in the MODY genes: HNF1A and HNF4A. J Clin Endocrinol Metab.

[7] Hamilton AJ, Bingham C, McDonald TJ (2014). The HNF4A R76W mutation causes atypical dominant Fanconi syndrome in addition to a beta cell phenotype. J Med Genet.

[8] Anyiam O, Wallin E, Kaplan F et al (2019) A complicated pregnancy in an adult with HNF4A p.R63W-associated Fanconi syndrome. Case Rep Med 2019:2349470. 10.1155/2019/2349470 10.1155/2019/2349470 PMC6944970 31949432

[9] Clemente M, Vargas A, Ariceta G et al (2017) Hyperinsulinaemic hypoglycaemia, renal Fanconi syndrome and liver disease due to a mutation in the HNF4A gene. Endocrinol Diabetes Metab Case Rep 2017:16-0133 10.1530/EDM-16-0133 PMC5404475 28458902

[10] Sheppard SE, Barrett B, Muraresku C, McKnight H, De Leon DD, Lord K, Ganetzky R (2021) Heterozygous recurrent HNF4A variant p.Arg85Trp causes Fanconi renotubular syndrome 4 with maturity onset diabetes of the young, an autosomal dominant phenocopy of Fanconi Bickel syndrome with colobomas. Am J Med Genet A 185(2):566–570. 10.1002/ajmg.a.61978 10.1002/ajmg.a.61978 PMC8132289 33251707

[11] Duan N, Huang C, Pang L, Jiang S, Yang W, Li H (2021) Clinical manifestation and genetic findings in three boys with low molecular Weight Proteinuria - three case reports for exploring Dent Disease and Fanconi syndrome. BMC Nephrol 22(1):24. 10.1186/s12882-020-02225-6 10.1186/s12882-020-02225-6 PMC7802264 33430795

[12] McGlacken-Byrne SM, Mohammad JK, Conlon N, Gubaeva D, Siersbæk J, Schou AJ, Demirbilek H, Dastamani A, Houghton JAL, Brusgaard K, Melikyan M, Christesen H, Flanagan SE, Murphy NP, Shah P (2022) Clinical and genetic heterogeneity of HNF4A/HNF1A mutations in a multicentre paediatric cohort with hyperinsulinaemic hypoglycaemia. Eur J Endocrinol 186(4):417–427. 10.1530/EJE-21-0897 10.1530/EJE-21-0897 35089870

[13] Brichta C, Pohl M, Lausch E, Kohlhase J, van der Werf-Grohmann N, Wurm N, Krause A, Schwab KO (2015) P3-1060 transient congenital hyperinsulinismm and renal Fanconi Syndrome. Horm Res Paediatr 84(S1):503

[14] Numakura C, Hashimoto Y, Daitsu T, Hayasaka K, Mitsui T, Yorifuji T (2015) Two patients with HNF4A-related congenital hyperinsulinism and renal tubular dysfunction: A clinical variation which includes transient hepatic dysfunction. Diabetes Res Clin Pract 108(3):e53-5. 10.1016/j.diabres.2015.03.005 10.1016/j.diabres.2015.03.005 25819479

[15] Improda N, Shah P, Güemes M, Gilbert C, Morgan K, Sebire N, Bockenhauer D, Hussain K (2016) Hepatocyte nuclear factor-4 alfa mutation associated with hyperinsulinaemic hypoglycaemia and atypical renal Fanconi syndrome: expanding the clinical phenotype. Horm Res Paediatr 86(5):337–341. 10.1159/000446396 10.1159/000446396 27245055

[16] Walsh SB, Unwin R, Kleta R, Van't Hoff W, Bass P, Hussain K, Ellard S, Bockenhauer D (2017) Fainting Fanconi syndrome clarified by proxy: a case report. BMC Nephrol 18(1):230. 10.1186/s12882-017-0649-8 10.1186/s12882-017-0649-8 PMC5504823 28693455

