# Supplementary material for: De novo HNF4A-associated atypical Fanconi renal tubulopathy syndrome
Source: J Nephrol. 2023 Jun 13;37(1):191–7. doi: 10.1007/s40620-023-01666-0 (PMC10920409; doi:10.1007/s40620-023-01666-0)
Supplement: Supplementary file 1 — Supplementary file1 (DOCX 1156 KB) [file 40620_2023_1666_MOESM1_ESM.docx]

**SUPPLEMENTAL MATERIALS**

**Supplemental Article Text**

In addition to our reported case, we conducted a systematic literature review using the PubMed database from inception through to May 2022. The search strategy used the terms “HNF4A,” “kidney” and “renal” combined with the Boolean operator “AND”. Full texts were retrieved and reviewed independently by two individual authors for inclusion. The search identified 24 cases^5-9, S10-S12, S14-S17^ of *HNF4A*-FRTS4 between 2012 and 2022, the clinical and laboratory data of these cases and our case are summarized in Table 1.

**Supplemental Methods**

*Literature Review*

We performed a literature search in May 2022 using the PubMed database from inception through to the 31^st^ of May, 2022. The search strategy used the terms “HNF4A,” “kidney” and “renal” combined with the Boolean operator “AND”. Full texts were retrieved and reviewed independently by two individual authors for inclusion. Articles were included if they specifically referenced the renal presentation of Fanconi syndrome and were written in English. Articles were excluded if they did not contain case studies, were conducted in non-human subjects or were not related to the presentation of atypical Fanconi syndrome or a near-synonymous phenotype. Reference lists of included articles were scanned for additional potential articles for inclusion. Following the compilation of included articles, data were extracted by an individual author on demographics, clinical presentation and investigations performed. The search strategy identified 75 citations, of which 11 were included for full text review. The cases from Britcha et al ^S^^15^ were identified following a further search on google scholar which identified a published poster presentation. Review of the literature was last performed in 2018 by Liu et al ^1^. The individual cases are summarised in Table 1. The years of publication ranged from 2012 to 2022. Publications originated from 5 countries, with the most common being the United Kingdom (n=5), there were 2 publications from China and the United States of America, and 1 publication from Spain, Germany and Japan, respectively. Publications were single case studies or case series.

*Research and Clinical Genomics*

The Royal Brisbane and Women’s Hospital (RBWH) Human Research and Ethics Committee (approval HREC/14/QRBW/34), the UQ Medical Research Ethics Committee (approval 2014000453) and the Children’s Health Queensland Human Research and Ethics Committee (approval HREC/15/QRCH/126), reviewed and approved the study. Research governance approval was also attained from the RBWH (SSA/14/QRBW/182) and Queensland Children’s Hospital (SSA/16/QRCH/116)^S^^25^. Seventy-nine families have been recruited to this study to date across a broad variety of kidney phenotypes, clinical diagnoses and proposed inheritance patterns. The family reported here had a presumed Dent Disease-like syndrome and negative diagnostic genetic testing as identified from the Queensland Conjoint Renal Genetics Clinics. Prior negative diagnostic genetic testing had been performed at the Children’s Hospital at Westmead applying virtual renal gene panels for analysis with targeted clinical exome (TruSight One) sequencing ^S^^26^.

Samples (2x5ml EDTA tubes of peripheral whole blood) were acquired after informed consent by trained phlebotomists. DNA extraction was undertaken in the Pathology Queensland PCR Core using an automated QIASymphony methodology from received whole blood samples. Whole genome sequencing was undertaken on an Illumina X instrument at the Garvan Institute (Darlinghurst, Australia). Reads were aligned to the reference human genome (GRCh37) using BWA-mem. single nucleotide variants (SNVs) and indels called using GATK v3.6, copy number variants (CNVs) identified with Canvas and Manta ^S^^27-S29^ . All variants were annotated using SnpEff v3.4.

Analysis and identification of candidate variants was performed with an in-house workflow incorporating the annotated variant data and pedigree information. Measures included in silico damage prediction (SIFT, Polyphen, LRT, MutationTaster/Assessor, FATHMM, PROVEAN, CADD), assessment of allele frequencies (1000Genome, ESP6500 and gnomAD databases^S^^30^), read quality analysis, and determination of pedigree concordance for all hypothesized inheritance patterns.

Sanger confirmation of candidate variants and independent assessment of variant pathogenicity and likely causality was performed by the accredited diagnostic molecular genetic laboratory at the Children’s Hospital at Westmead as a component of standard clinical care. Assessment of variant pathogenicity and likely causality for the observed phenotypes was independently assessed by the diagnostic laboratory according to American College of Medical Genomics (ACMG) clinical genetic variant assessment and reporting guidelines. The genetic findings were reported back to the participants’ clinical geneticist and nephrologist for clinical disclosure including further testing and additional genetic counselling.

**Supplemental References**

S10. Improda N, Shah P, Guemes M*, et al.* Hepatocyte Nuclear Factor-4 Alfa Mutation Associated with Hyperinsulinaemic Hypoglycaemia and Atypical Renal Fanconi Syndrome: Expanding the Clinical Phenotype. *Horm Res Paediatr* 2016; **86:** 337-341.

S11. Numakura C, Hashimoto Y, Daitsu T*, et al.* Two patients with HNF4A-related congenital hyperinsulinism and renal tubular dysfunction: A clinical variation which includes transient hepatic dysfunction. *Diabetes Res Clin Pract* 2015; **108:** e53-55.

S12. Walsh SB, Unwin R, Kleta R*, et al.* Fainting Fanconi syndrome clarified by proxy: a case report. *BMC Nephrol* 2017; **18:** 230.

S13. Pieck AV, Leon MC, Martorell AC*, et al.* Congenital hyperinsulinaemic hypoglycaemia of infancy, renal Fanconi syndrome and hepatopathy due to a mutation in the HNF4A gene. *ESPE Abstracts* 2015; **84**.

S14. Brichta CM, Pohl M, Lausch E*, et al.* Transient Congenital Hyperinsulinism and Renal Fanconi Syndrome. *ESPE Abstracts* 2015; **84**.

S15. Duan N, Huang C, Pang L*, et al.* Clinical manifestation and genetic findings in three boys with low molecular Weight Proteinuria - three case reports for exploring Dent Disease and Fanconi syndrome. *BMC Nephrol* 2021; **22:** 24.

S16. McGlacken-Byrne SM, Mohammad JK, Conlon N*, et al.* Clinical and genetic heterogeneity of HNF4A/HNF1A mutations in a multicentre paediatric cohort with hyperinsulinaemic hypoglycaemia. *Eur J Endocrinol* 2022; **186:** 417-427.

S17. Sheppard SE, Barrett B, Muraresku C*, et al.* Heterozygous recurrent HNF4A variant p.Arg85Trp causes Fanconi renotubular syndrome 4 with maturity onset diabetes of the young, an autosomal dominant phenocopy of Fanconi Bickel syndrome with colobomas. *Am J Med Genet A* 2021; **185:** 566-570.

S18. Sladek FM, Ruse MD, Jr., Nepomuceno L*, et al.* Modulation of transcriptional activation and coactivator interaction by a splicing variation in the F domain of nuclear receptor hepatocyte nuclear factor 4alpha1. *Mol Cell Biol* 1999; **19:** 6509-6522.

S19. Betcherman L, Lemaire M, Licht C*, et al.* Helping nephrologists find answers: hyperinsulinism and tubular dysfunction: Answers. *Pediatr Nephrol* 2020; **35:** 257-260.

S20. Xanthopoulos KG, Prezioso VR, Chen WS*, et al.* The different tissue transcription patterns of genes for HNF-1, C/EBP, HNF-3, and HNF-4, protein factors that govern liver-specific transcription. *Proceedings of the National Academy of Sciences* 1991; **88:** 3807-3811.

S21. Marchesin V, Perez-Marti A, Le Meur G*, et al.* Molecular Basis for Autosomal-Dominant Renal Fanconi Syndrome Caused by HNF4A. *Cell Rep* 2019; **29:** 4407-4421 e4405.

S22. Kobayashi A, Valerius MT, Mugford JW*, et al.* Six2 defines and regulates a multipotent self-renewing nephron progenitor population throughout mammalian kidney development. *Cell Stem Cell* 2008; **3:** 169-181.

S23. Marable SS, Chung E, Adam M*, et al.* Hnf4a deletion in the mouse kidney phenocopies Fanconi renotubular syndrome. *JCI Insight* 2018; **3**.

S24. Uhlen M, Fagerberg L, Hallstrom BM*, et al.* Proteomics. Tissue-based map of the human proteome. *Science* 2015; **347:** 1260419.

**SUPPLEMENTAL FIGURES**

**Supplementary Figure 1.** Kidney biopsy at age 12 years. Light microscopy picture (magnification x400) reveals minor tubular epithelial changes including epithelial flattening and apical blebbing.


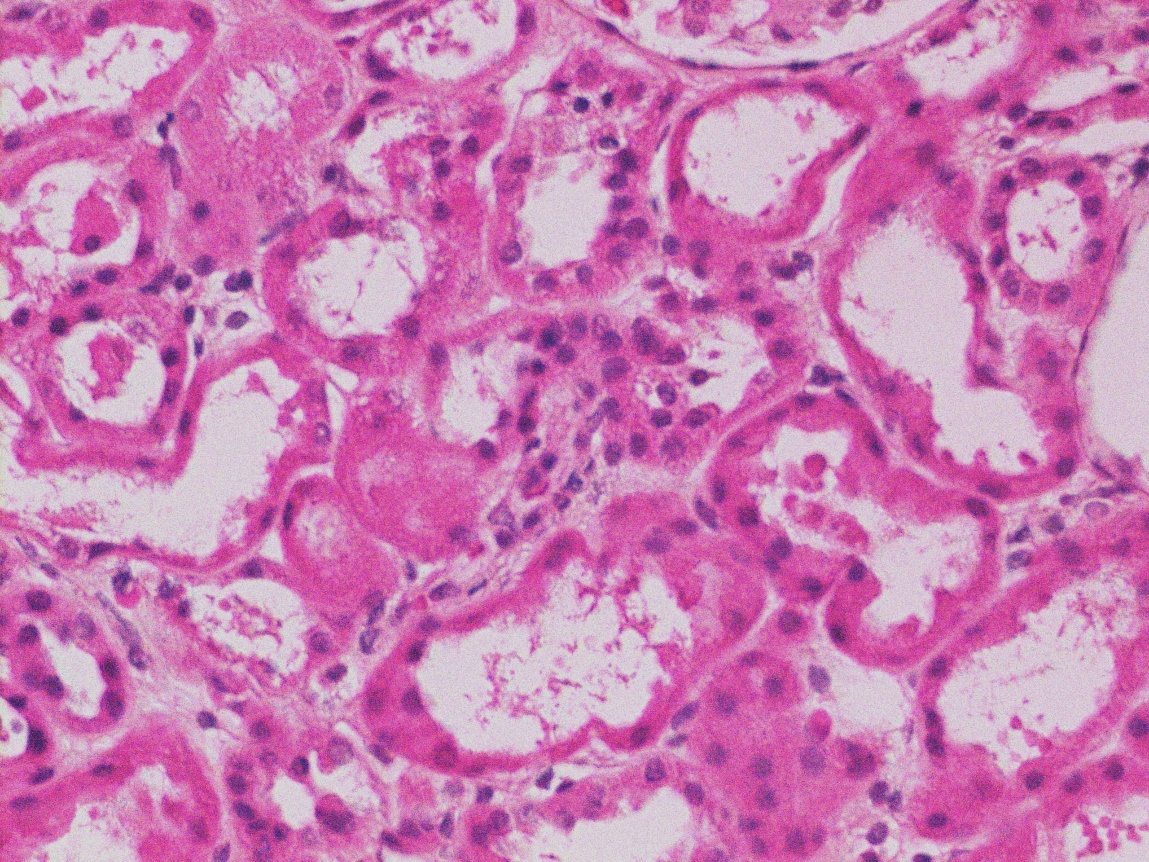


**Supplementary Figure 2.** Kidney biopsy at age 12 years. Electron microscopy picture (magnification x10,000) of a proximal tubular epithelial cell. There are some abnormalities in the morphology of the mitochondria including; derangement of cristae, vacuolation and reduction in the size and number of granules.


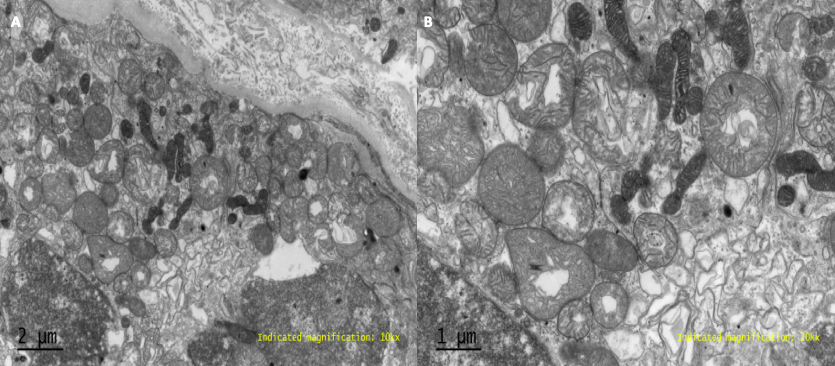


**Supplementary Figure 3.** (3A) X-ray of the left femur revealing delayed union of the midshaft fracture. (3B) X-ray of the left tibia and fibula revealing a healed fracture of the tibia and a visible fracture line in the fibula, indicating delayed union. This case required multiple multi-level orthopaedic procedures due to his hypophosphataemic rickets.
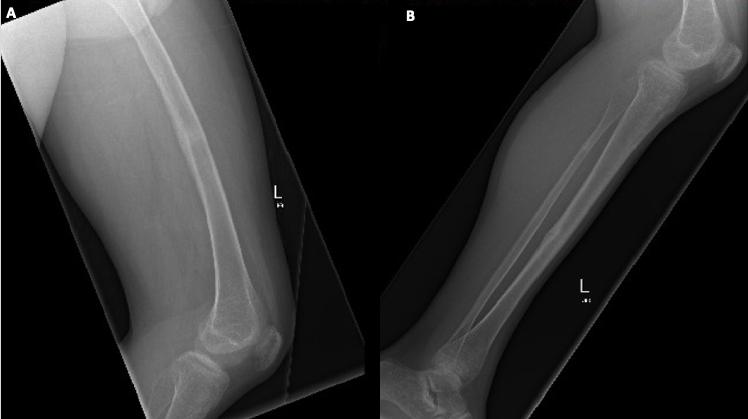


**SUPPLEMENTARY TABLE**

|  | Age | Creatinine | GFR (estimated) | Potassium | Bicarbonate | Glucose | Calcium (Alb. Corr.) | Phosphate | Magnesium | Albumin | Alkaline Phosphatase | Urine PCR | ACR | Urinary calcium | PTH | Timed urinary phosphate | Timed urinary calcium | Timed urinary creatinine | Urinary amino acids | Urinary glucose |
| --- | --- | --- | --- | --- | --- | --- | --- | --- | --- | --- | --- | --- | --- | --- | --- | --- | --- | --- | --- | --- |
| Units |  | μmol/L | mL/min/1.73m^2^ | mmol/L | mmol/L | mmol/L | mmol/L | mmol/L | mmol/L | g/L | U/L | g/mol | mg/L | mmol/l | pmol/L | (mmol/24h | mmol/24h | mmol/L | y/n | mmol/l |
| Range |  | 60-100 | >90 | 3.5-5.2 | 22-32 | 3.0-7.8 | 2.10-2.60 | 0.75-1.50 | 0.70-1.10 | 35-50 | 30-110 | <15 | <1.0 | - | 1.0-7.0 | 11.0-32.0 | 2.5-7.5 | - | - |  |
|  | 26 | 166 |  | 3.7 | 19 | 11.5 | 2.27 | 0.78 | 1 | 45 | 137 |  | 307 |  |  |  |  |  |  |  |
|  | 25 | 175 | 46 | 3.7 | 22 | 10.8 | 2.15 | 0.71 | 1.12 | 43 | 130 | 240 | 120 |  |  |  |  |  |  |  |
|  | 24 | 212 | 37 | 3.8 | 22 | 10.6 | 2.25 | 0.82 | 1.04 | 47 | 172 | 285 | 87 |  |  |  |  |  |  |  |
|  | 23 | 140 | 61 | 4.1 | 20 | 7.3 | 2.06 | 0.63 | 1.17 | 47 | 188 | 292 | 120 |  | 16 |  |  |  |  |  |
|  | 22 | 147 | 58 | 3.8 | 21 | 18.2 | 2.02 | 0.69 | 1.27 | 46 | 257 | 232 | 100 |  | 22 |  |  |  |  |  |
|  | 21 | 136 | 64 | 3.7 | 23 | 6.9 | 2.2 | 0.6 | 1.18 | 44 | 231 | 232 | 202 |  | 5.1 |  |  |  |  |  |
|  | 20 | 135 | 65 | 3.9 | 23 | 5.1 | 2.17 | 0.52 | 1.27 | 45 | 157 | 252 | 72 |  | 7.1 | 34.4 | 5.7 | 11.1 | Yes | 165 |
|  | 19 | 151 |  | 3.4 | 22 |  | 2.01 | 0.73 | 1.16 | 45 | 336 |  |  |  |  |  |  |  |  |  |
|  | 18 |  |  |  |  |  |  |  |  |  |  |  |  |  |  |  |  |  |  |  |
|  | 17 | 123 |  | 4.6 | 22 | 6.6 | 2.08 | 0.73 |  | 48 | 583 | 264 | 31 | 1.2 | 46 |  |  |  |  |  |
|  | 6 | 93 |  | 3.4 | 19 | 5.8 | 2.23 | 0.91 | - | 46 | 518 |  |  |  |  |  |  |  |  |  |

**Supplementary Table 1:** Serum and biochemical data for case patient from age 6 to 26 years old. Available data has been collated where accessible
